# The effect of shape and size in the stability of triangular Janus MoSSe quantum dots

**DOI:** 10.1038/s41598-021-00287-6

**Published:** 2021-10-26

**Authors:** J. I. Paez-Ornelas, R. Ponce-Pérez, H. N. Fernández-Escamilla, D. M. Hoat, E. A. Murillo-Bracamontes, María G. Moreno-Armenta, Donald H. Galván, J. Guerrero-Sánchez

**Affiliations:** 1 Centro de Investigación Científica y de Educación Superior de Ensenada, Ensenada Baja California, 22800 Mexico; 2 Centro de Nanociencias y Nanotecnología, Universidad Nacional Autónoma de México, Apartado Postal 14, Ensenada Baja California, 22800 Mexico; 3 Institute of Theoretical and Applied Research, Duy Tan University, Hanoi, 100000 Viet Nam; 4 Faculty of Natural Sciences, Duy Tan University, Da Nang, 550000 Viet Nam

**Keywords:** Materials for energy and catalysis, Nanoscale materials

## Abstract

Asymmetric Janus transition metal dichalcogenide MoSSe is a promising catalytic material due to the intrinsic in-plane dipole of its opposite faces. The atomic description of the structures observed by experimental techniques is relevant to tuning and optimizing its surface reaction processes. Furthermore, the experimentally observed triangular morphologies in MoSSe suggest that an analysis of the chemical environment of its edges is vital to understand its reactivity. Here we analyze the size-shape stability among different triangular structures-quantum- dots proposed from the ideal S(-1010) and Mo(10-10) terminations. Our stability analysis evidenced that the S–Se termination is more stable than Mo; moreover, as the size of the quantum dot increases, its stability increases as well. Besides, a trend is observed, with the appearance of elongated Mo-S/Se bonds at symmetric positions of the edges. Tersoff–Hamann scanning tunneling microscopy images for both faces of the stablest models are presented. Electrostatic potential isosurfaces denote that the basal plane on the S face of both configurations remains the region with more electron density concentration. These results point toward the differentiated activity over both faces. Finally, our study denotes the exact atomic arrangement on the edges of MoSSe quantum dots corresponding with the formation of S/Se dimers who decorates the edges and their role along with the faces as catalytic sites.

## Introduction

Transition metal dichalcogenides (TMDs) are nowadays among the most investigated and characterized materials in solid-state physics. The amply different features shown by the bulk and the monolayer (ML) have led to a broad scope of applications in diverse areas such as nanoelectronics^1,2^, energy storage^3^, spintronics^4,5^, and catalysis^6^.

TMDs have a general formula $MX_2$ where M is a transition metal, and X represents a chalcogen element. The bulk TMDs are made up of stacked laminar structures through van der Waals-type interactions where each layer is composed of three species (X-M-X)-forming a single triple layer. This fact has allowed ML isolation by cleavage or mechanical exfoliation^7^. Moreover, interesting properties emerge during the shift from bulk to ML phases: transitioning from indirect to direct bandgap^8^, spin-valley coupling^9^, and good carrier mobility at room temperature, renewing the interest in the 2D layered TMD family^10^.

In TMDs, the transition metal coordination with the chalcogen elements and the stacking order can lead to four differentiated phases: 1T, and distorted 1T’ with octahedral coordination, plus 2H and 3R with trigonal prismatic coordination^11^. However, in the ML, the dominant phases are 1T, and 1H-a single triple-layer from 2H bulk^12^. These structures have been the focus of intense research as they present intrinsic in-plane asymmetry^13^. The asymmetry is vital since, compared to other laminar materials such as graphene leads to obtaining direct bandgap materials without introducing external fields or layer stacking^14^. Their electronic behavior has been a key factor to be considered active participants in catalytic reactions^15^.

The performance of TMDs ML as catalytic materials has been demonstrated during the last century with the use of $MoS_2$ to catalyze several petroleum refinery reactions as hydrodesulfurization (HDS) hydrogenation (HYD), and metals remotion. However, the details of its atomic structure were unknown until the Besenbacher group obtained the first STM images^16^. Their study concluded that the ML of $MoS_2$ stabilizes as triangular quantum dots of varied sizes under sulfiding conditions. So far, many combinations of TMDs have been investigated in the hunt for materials with enhanced catalytic activity.

Recently, a structural modification has achieved an additional degree of freedom in TMD ML, causing out-of-plane asymmetry to form the so-called Janus ML^17^. This family has the general formula XMY and can be obtained by the substitution of one chalcogen atom of the single triple-layer^18^. Since X and Y are different chalcogen elements, the surfaces are expected to possess distinctive electronic behavior and different catalytic activity.

Although different Janus structures have been theoretically predicted as stable^19–21^, only a few reports of its experimental synthesis exist. The groups of Li and Lou have reported two different procedures to synthesize the 1H MoSSe Janus ML^22,23^.

In the methodology presented by Li^22^, a $MoSe_2$ ML template was grown by CVD. Direct exchange of the top layer was performed by a controlled substitution reaction of S in the $MoSe_2$ ML to form MoSSe. On the other hand, a $MoS_2$ ML template was used in Lou’s methodology. The $MoS_2$ ML was exposed to $H_2$ plasma to remove the top S layer and form MoSH. Finally, a selenization of the sample is reached with thermal vaporization of Se powders to obtain MoSSe. The structures obtained were analyzed by STM. Their morphology is consistent with triangular quantum dots of diverse dimensions as also observed in $MoS_2$ STM investigations^16,24^. However, there is not enough structural information to build an exact atomic model that allows studies on the edge terminations of these structures, the stability, and the role in catalytic reactions^25^. Further analysis is needed to provide further insight into morphology and border-termination; such atomic-scale description may help understand and improve materials with enhanced catalytic activity.

This article discusses the atomic arrangement observed in Li and Jun’s experimental pioneering work to synthesize 1H MoSSe Janus ML. We study the stability by first principles-thermodynamics of four triangular structures consistent with the experimentally observed morphology, which may have Mo or S–Se termination. Our results denote that the configurations with S/Se dimers on edge remain the most stable models. Furthermore, from a structural analysis for triangles of different sizes, a trend is observed by the appearance of elongated Mo-S/Se bonds in symmetric positions of the edge for large triangles. Tersoff-Hamann scanning tunneling microscopy simulations of the stablest model are presented. Electrostatic potential isosurfaces point that the basal plane on the S face of both configurations remains the region with higher electron density. Their role hints at the preferential activity of the faces. Finally, our study denotes the exact atomic arrangement on the edges of MoSSe quantum dots corresponding with S/Se dimers that decorate the edges and their role -along with the faces-as catalytic sites.

## Results

In this work, different triangular structures obtained via the ML breaking in the low index edge termination were proposed to study their stability as a function of size and edge termination.

First, the hexagonal 1H-MoSSe ML unit cell was optimized and used as a template from which the triangular structures were obtained. The calculated lattice parameter was $a_0=3.23$ Å in good agreement with the experimental value of 3.22 Å^22^, the Mo-S/Se bond distances were 2.41 and 2.53 Å respectively, whereas the angles $\angle$MoSMo, $\angle$SMoSe, $\angle$MoSeMo had the values: 83.98$^\circ$, 81.92$^\circ$, and 79.35$^\circ$.

As mentioned in the “Methods” section, the reference set for this study has been the ${MoS_2}^{Bulk}$; therefore, we calculated the formation enthalpy ($\Delta H_f$) for this system, obtaining a value of 2.58 eV in accordance with previous theoretical calculations^26,27^, and very close to the experimental value of 2.86 eV^28^.

### Structural configurations

In light of the morphology observed through experimental synthesis^22,23^, we focus on the equilibrium shape of two-dimensional triangular quantum dots. Interestingly, the triangular shape suggests that only one edge type must be energetically favored^24^. This fact is consistent with the Wulff construction argument^29^ where only one of the two low index edge terminations must predominate-the Mo $(10{\bar{1}}0)$ or S/Se $({\bar{1}}010)$ edges^16,24,30^. However, the role of edge atoms may be absent when using this classical construction; therefore, we dedicate a discussion of the role of edge atoms in minimizing the energy of the most stable models following the generalized Wulff construction^31^.

**Figure 1:**
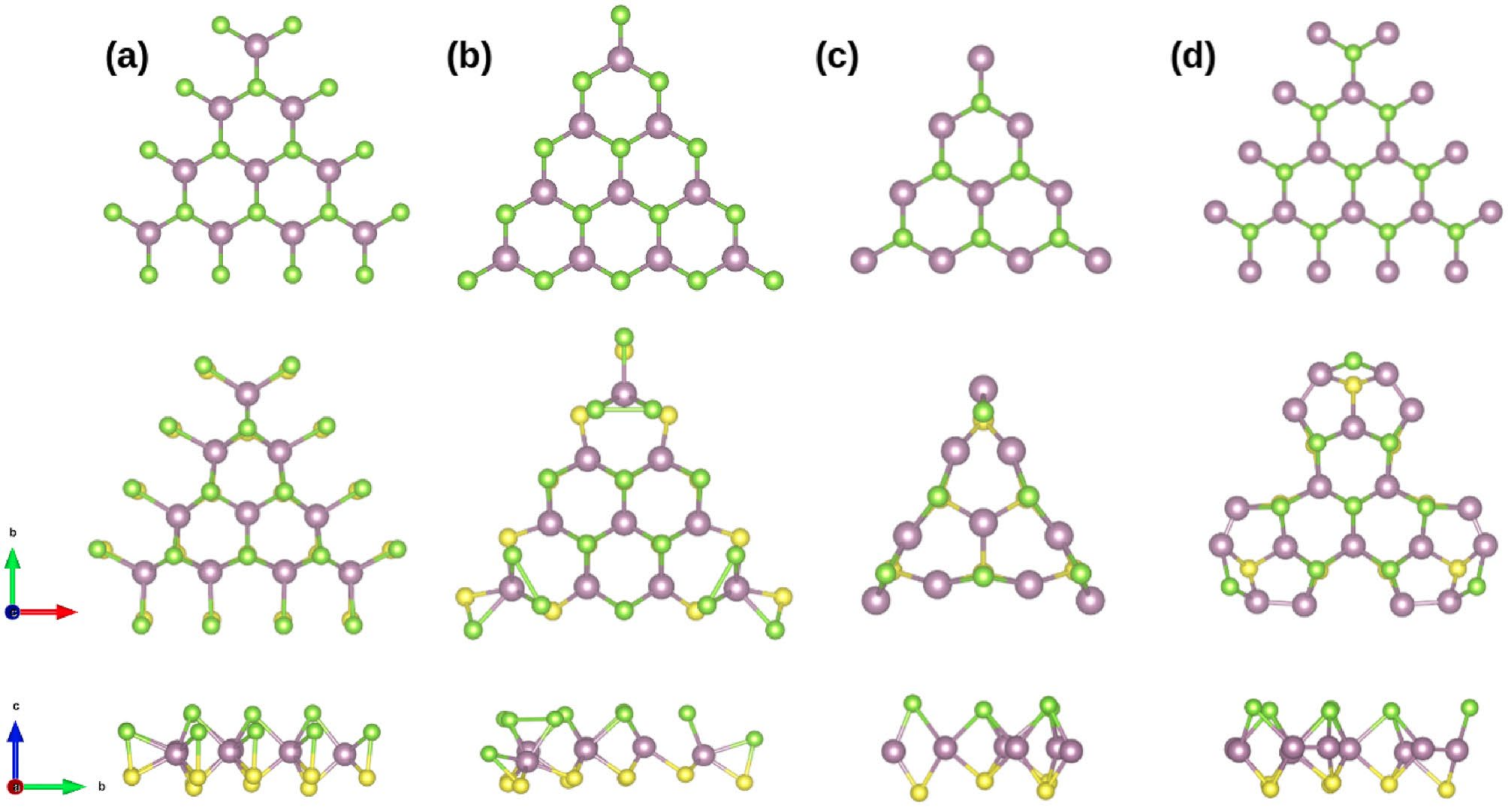
1H-MoSSe quantum-dots obtained by the breaking of the ML in the low symmetry indexes direction. (**a**) C1(4) from the $(10{\bar{1}}0)$ termination depicts Mo atoms in six-fold coordination where each Mo over the edge is bonded to a protruding S/Se dimer. (**b**) C2(4) from the $({\bar{1}}010)$ termination, where the Mo atoms are also six-fold coordination. However, the Mo atoms from each edge are bonded with two S/Se dimers forming a zig-zag pattern. (**c**) C3(4) where S/Se atoms are three-fold coordinates with the Mo atoms forming a zig-zag edge with Mo exposed, and (**d**) C4(4) obtained from C1 exchanging Mo atoms with S/Se dimers. S, Se, and Mo atoms are depicted as yellow, green, and violet spheres.

The triangular structures were built based on the Mo $(10{\bar{1}}0)$ or S/Se $({\bar{1}}010)$ terminations from the MoSSe ML. Since both terminations present different exposure of the border atom (see Fig. 1a–d), four different structures can emerge: two with S–Se dimers as the protruding species labeled as C1 and C2 where each Mo atom is six-fold coordinated, plus other two configurations with protruding Mo atoms labeled as C3 and C4 where the S and Se atoms of each face are three-fold coordinated with the closest Mo atoms. In these configurations, the coordination of the species except for the border atoms resembles that of the ML.

Note that the ideal structures (see the upper part on Fig. 1) contain the same number of Mo atoms per edge; this number is employed to set the size of the system under study. Therefore, we constructed models for the four configurations containing n Mo atoms per edge where n can take values ranging from 4 to 10. Depending on the configuration, these systems are labeled as CX(n).

In C1, the $(10{\bar{1}}0)$ termination contains S–Se protruding dimers where each dimer is bonded with a single Mo atom over the edge in a triangular area enclosed by the Mo atoms (see Fig. 1a). After structural relaxation, this configuration remains less distorted; note that the S–Se dimers over the edge tend to stabilize the deformation of the structure. The S–Se bond distance is 2.13 Å in the inner part of the edges and 2.17 Å on the corners.

Subsequently, C2 obtained from the $({\bar{1}}010)$ termination is characterized by the formation of a zig-zag edge where the S–Se dimers protrude and are bonded with two adjacent Mo atoms (see Fig. 1b). Note that after relaxation, in each corner over the Se side of the Janus structure, Se dimers tend to appear over the Mo atom of the corner with bond distance ranging between 2.44 and 2.46 Å. The side view shows that this structure tends to curve towards the face of S atoms. This behavior becomes more remarkable as the size of the system increases.

The exposure of Mo atoms characterizes the following configurations and indeed are those that present a greater structural distortion (see the lower part on Fig. 1c). C3 corresponds to a $(10{\bar{1}}0)$ fully Mo terminated; note that this configuration corresponds to C1 with the remotion of the S/Se dimers over the edge. After structural relaxation, the Mo atoms on the edges tend to rearrange to dominate the Mo-Mo interaction; their displacement towards the center evidences this behavior. Finally, an additional configuration, C4, can be constructed by exchanging positions between S–Se dimers and Mo atoms from C2. This system is, in fact, the one that presents a greater atomic rearrangement (see Fig. 1d).

The edge terminations of these systems have been previously considered in detail only for the predecessor structure $MoS_2$. The Besenbacher group confirmed by STM images that the nanoparticles had triangular shapes with S terminations providing a new picture of reactivity in this class of structures^16^. Moreover, based on the fact that triangular morphologies suggest that one of the edge terminations must be more stable, other theoretical works have been devoted to determining the exact atomic arrangement over a wide range of sulfiding conditions^26^. The results presented through DFT by Joo et al.^26^ were consistent with the assumption of Besenbacher, where the edge protrusion of the triangular structures should be associated with S atoms whose height was shifted by a half lattice constant compared with the truncated position of the ML. It is important to mention that such a difference in height for the MoSSe structure was not identified through atomic force microscopy (AFM) measurements; in fact, the height was consistent with that of the monolayer, suggesting that the presence of two atomic species modifies the arrangement of the edges—this is Se and S^22^.

Nevertheless, no conclusion about the termination and stability of the MoSSe system has been developed. Therefore, we present a stability analysis that compares the previously described structures and simple Mo or combined S/Se termination role.

### Stability

Next, once we explain the triangular configurations that can be obtained from the MoSSe ML, we have employed the surface formation energy formalism to analyze their formation energy accordingly to Eq. (5) as for systems of the same size (n Mo per edge) the number of atoms is different.

**Figure 2:**
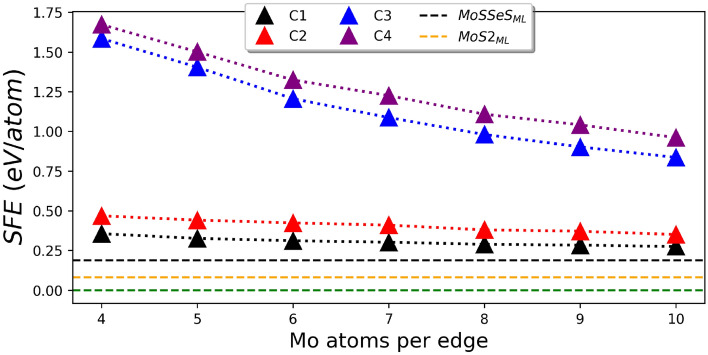
SFE for the four proposed triangular MoSSe quantum dots. C1, C2, C3, and C4 are represented by black, red, blue, and purple triangles. The $MoS_2^{Bulk}$ is set as the reference and is depicted as a dotted green line. Over the reference, the $MoS_2$ and MoSSe MLs are represented by an orange and black dotted line.

The calculated surface formation energy (SFE) (for details of the formalism see “Formation energy formalism”) of the proposed terminations: C1(n), C2(n), C3(n) and, C4(n) as a function of their size is depicted in Fig. 2. The size of the different configuration range between 4 and 10 Mo per edge. The $MoS_2^{Bulk}$ seated as the reference in our thermodynamic analysis is represented by a green dotted line. Since we are working with systems coming from the ML, we have introduced two additional references that appear slightly over the $MoS_2^{Bulk}$: this is the $MoS_2$ ML followed by the MoSSe ML. These systems are represented by dotted orange and black lines, respectively. Above these values, the systems under study are shown; note that as the values move away from the reference, their stability decreases; however, as these values tend to get closer to the reference, their stability increases, which indicates that their formation is likely to appear.

Our findings show that the S/Se terminated systems: C1(n) and C2(n) are more stable than Mo terminated: C3(n) and C4(n). Moreover, as the size of the system increases, a trend is observed where all systems tend to stabilize, approaching the *MoSSe* ML. Note that Mo terminated configurations present a more marked change in energy evidenced by an abrupter drop in their slopes. This behavior has been described for triangular structures. It can be correlated with two perspectives associated with the number of unsaturated atoms over the edge^26^. First, as the system size increases, the ratio of S/Se and Mo atoms increases to reach the bulk value. The second perspective considers the opposite trend. The ratio between the atoms over the edge and the total number of atoms decreases, indicating that the number of not fully coordinated Mo atoms over the edge is reduced. Therefore, an interplay related to an increment in the system size is evident to gain stability.

On the other hand, for the S/Se terminated systems, the stability trend is uniform, suggesting that structures throughout the full-size range have the same probability of appearing under experimental conditions. Note that C1(n) and C2(n) have a slight energy difference so that both terminations could coexist in all the ranges of size under experimental conditions^24^.

Note that C1 and C2 maintain the coordination number of the ML; this is vital since the Mo edge atoms retain their coordination number of six being bonded with three adjacent Mo/Se atoms. The main distinctions are that C2 tends to form three Se dimers on each tip of the structure and fewer S–Se dimers over the edges. Both structures are absent Mo d-orbitals dangling bonds. The fact that there are no unsaturated d orbitals on the edges minimizes structural rearrangement^31^. Even with the variation of the chemical potential for all growth conditions (see Fig. S1), C1 remains the most stable structure.

The size distribution of these structures has been examined before in $MoS_2$ quantum dots^24,32^. The analysis unveiled that after close inspection of the size distribution of triangles in STM images, two main groups of distribution could be set: for the larger triangles, it was determined that predominant size was related with the number of Mo atoms per edge: with n = 8, 10, 12, and 14, as the size of the system increased the species count decreased. In contrast, in smaller triangles, the trend was the opposite. The highest abundance corresponded to structures with n = 4 followed by n = 6 Mo per edge.

This analysis of the size of the system is vital since the dimensionality effect in the electronic properties of confined systems has been commonly established, resulting in differentiated and even outstanding catalytic activity in several reactions as hydrogen-evolution reaction (HER) or oxygen-reduction reaction (ORR) as a function not only of edge termination but also of the size^26,32^.

### Geometrical distortion

Motivated by the differentiated rearrangement over both faces of the Janus structures, we have performed a detailed bond distances study, both in the center of the structures and on the edges. Besides, as the system size increases, some edge regions present elongated Mo–S/Se bonds or the formation of new S/Se dimers. This fact must be monitored to understand the sites where S or Se could be shed during reactions catalyzed by these structures.

**Figure 3:**
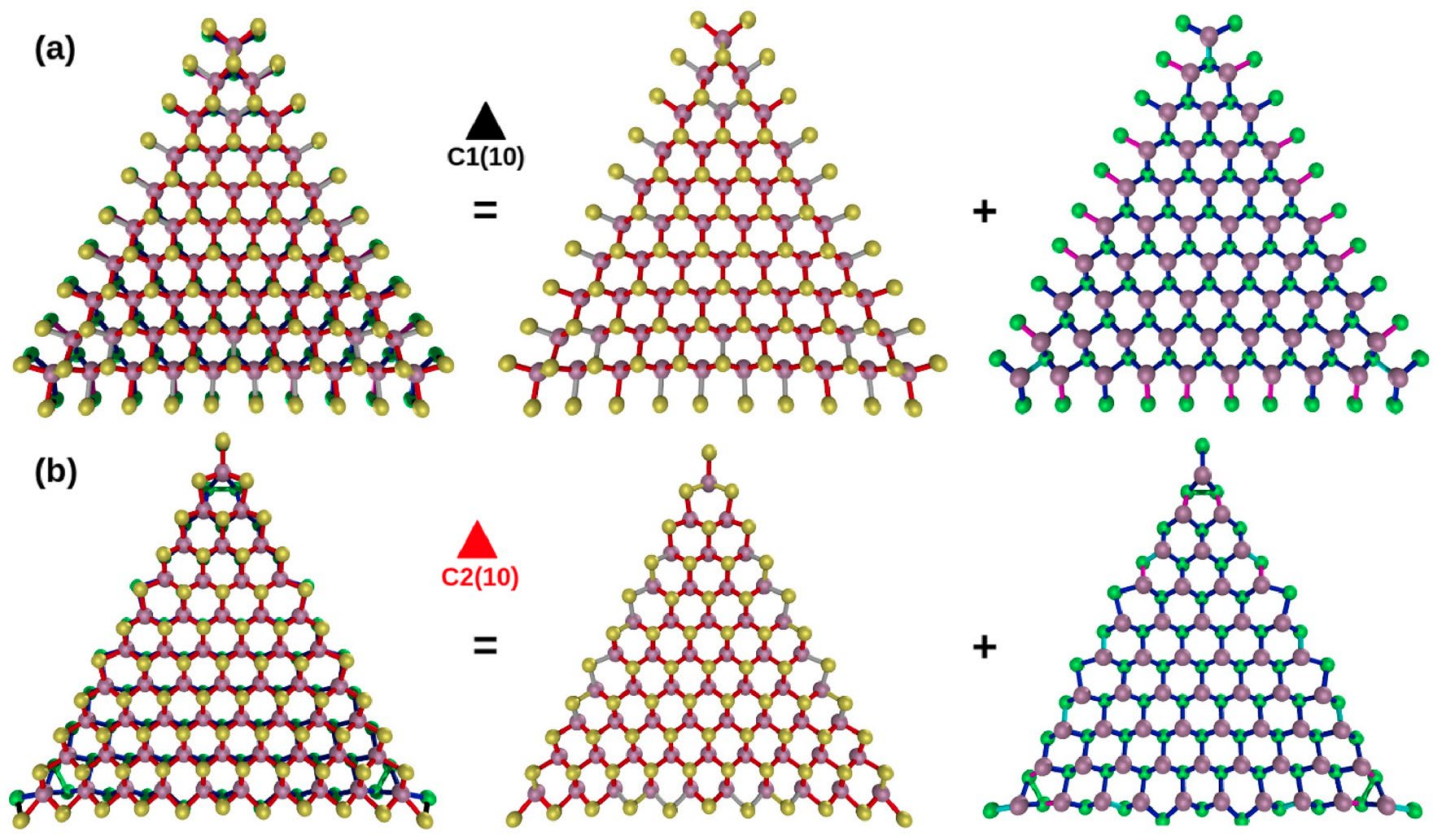
Bond length distribution of the stablest S/Se terminations. The image at the left is formed by the combination of adjacent images where each face is shown (**a**) C1(10) and (**b**) C2(10). S, Se, and Mo atoms are depicted as yellow, green, and violet spheres.

We have selected two model systems containing the broader bond distribution to show the bond distribution present in these structures. The chosen systems C1(10) and C2(10) are presented in Fig. 3 where a color code has been applied to differentiate between bond ranges.

For both configurations, a figure showing the distribution on both faces is presented. The individual contributions of the S or Se faces are shown on the right-hand side. Note that according to the color code for C1(10) (see Fig. 3a), the shorter bonds correspond with the S–Se dimers that decor the edges of the structure; these bonds range between 2.12 and 2.16 Å. Analyzing the S side of the Janus structure, the next bond in yellow corresponds to the Mo atom from the vertex and the S located towards the center of the structure. The length of this bond is highly uniform, with a value of 2.35 Å. The following bond in red color is associated with the Mo–S interaction ranging between 2.67 and 2.42 Å; the amply distribution presented by this bond is consistent with the fact that many S atoms from the edges undergo a structural rearrangement by moving away from the ideal value of 2.41 of the ML. Finally, the more elongated bonds with values among 2.42–2.45 Å are depicted in gray.

Now, the Se side shows a contracted Mo–Se bond with a value of 2.48 Å in cyan color. This bond is correlated with the Mo atom from the vertex and the adjacent Se atom that points toward the center. Next, the wider bond distribution is shown in blue, with values that range between 2.49 and 2.58 Å. Once again, using the previous analysis, it can be determined that this distribution corresponds to the structural rearrangement of the Se atoms at the edge. Finally, the broader bond with values between 2.58 and 2.61 Å is shown in magenta. Its main contribution is in the center of the edges and on symmetrical positions contiguous to the tips of the structure.

In C2(10), a wider variety of bond distances can be observed. As mention before, the concavity of the C2(n) is greater than in C1(c), therefore as the structure tends to bend, the bonds elongation(contraction) of the Se(S) is more evident as the system size increases. In this type of termination, two types of dimers appear in the color code scheme (see Fig. 3b): S–Se in black, that can be observed at each vertex, but also two additional dimers are formed over each edge, these dimers locate next to the S–Se at the edge center with a bond distance of 2.21–2.22 Å. The other class of dimers in green is Se-Se, located over each tip with a distance of 2.43–2.45 Å. It is relevant to mention that for systems with $n<9$ the appearance of a single S–Se dimer is observed on edge, while for $n=10$ an additional dimer is formed.

From the S side, the next bond in yellow is the Mo–S with a value of 2.25–2.29 Å; note that their location corresponds to the Mo of the vertex, and the concavity of the structure forms a pair of these on the fourth and fifth Mo atom over each edge. The rearrangement of the electronic distribution on the highlighted Mo atoms creates, as a related consequence, the appearance of an elongated Mo–S bond; this bond depicted in gray has the longest value of 2.45–2.46 Å. This last type of elongated bond is observed only in structures with $n>7$; for smaller triangles, such elongation does not exist. Finally, the remaining Mo–S bonds are displayed in red; the distribution reflects the noticeable structural changes over the edges; these bonds values are in the 2.30–2.42 Å range.

Concerning the Se side, the first kind of bond that appears is the Se dimer in green described previously. Next in length, the Mo-Se interaction can be classified into three groups; the smaller distance with a value of 2.45–2.48 Å is shown in cyan, located at the vertex of the structure and over the third and fifth Mo atom. The next bond in blue corresponds to bonds in the 2.49–2.6 Å range; as can be observed, this value oscillates around the ideal Mo-Se value of 2.53 Å. Finally, the longer bond of 2.61–2.65 Å is shown in magenta.

**Figure 4:**
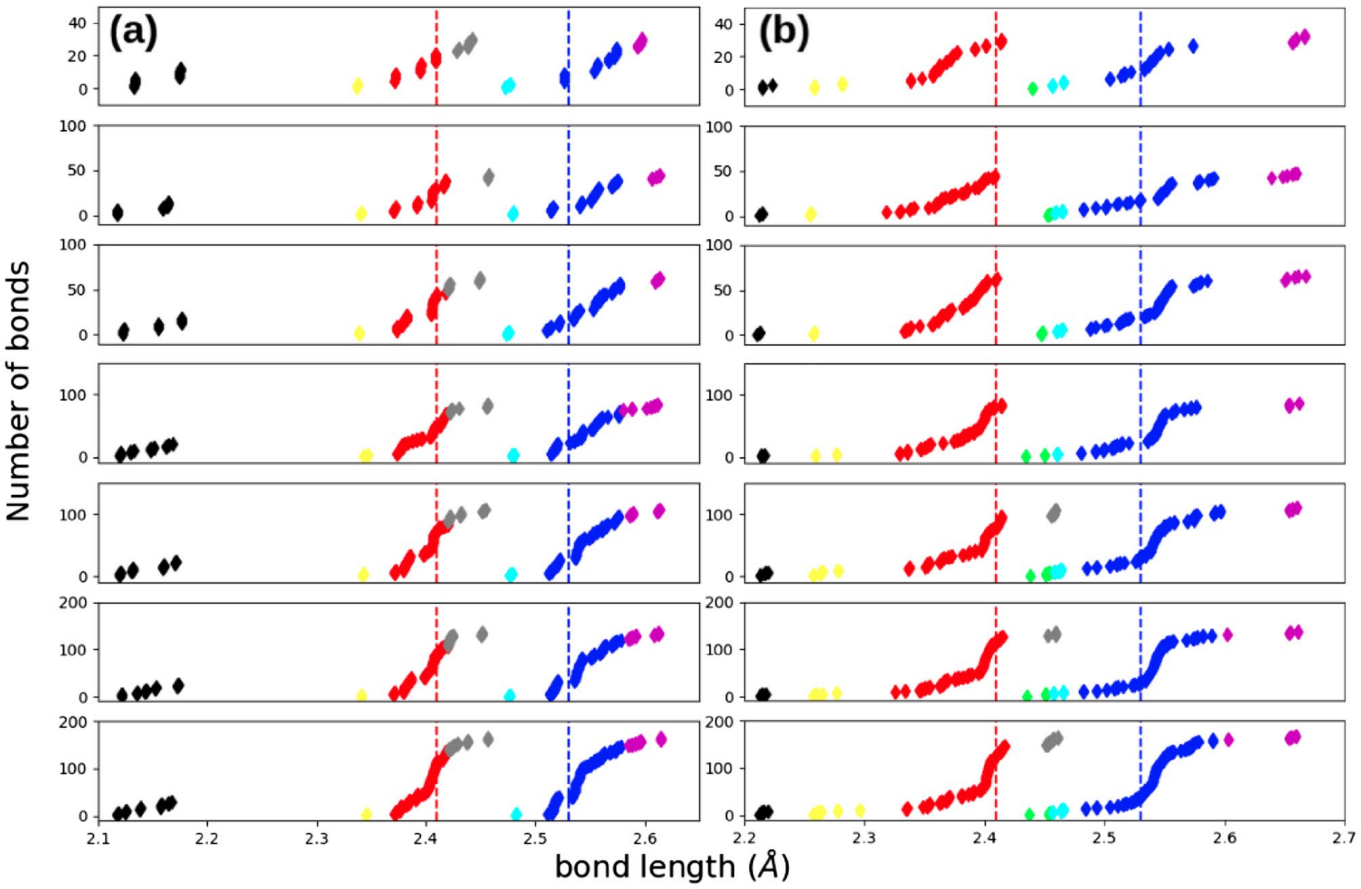
Bond distance distribution for the stablest S/Se terminated configurations. (**a**) C1(n) with n = 4–10 from top to bottom, and (**b**) C2(n) with n = 4–10 from top to bottom.

A bond length analysis, as reported before, has been applied to the S/Se terminated configurations C1 and C2 to study the morphological differences over the edges and the center of the structures; these results are summarized in Fig. 4. The configurations shown from top to bottom correspond to the system size from n = 4 to 10. The ideal values of the Mo-S(Se) bonds of 2.41(2.53)Å are represented by a vertical dotted line in red(blue). The color code employed in the previous section is kept to show the number of bonds against the bond distance.

The S–Se dimers represented by black diamonds are located in the left part of both graphs. For C1(n) (see Fig. 4a), as the size of the system increases, the formation of new dimers around the structure is evident; the bond measures between these are varied to support the structural rearrangement towards the edges. In contrast, C2(n) (see Fig. 4b) present a uniform distribution since the S–Se dimer is only located at the corners. Nevertheless, as mentioned, for systems with $n>7$, the appearance of new points can be perceived; these values are similar in length. The S–Mo bond of the tips in yellow is again uniform for C1(n) since it is associated exclusively with the Mo of the vertex. In contrast, for C2(n), the system size causes the appearance of new contracted bonds related to the S–Mo bond at the edges; again, as the system increase in size, a wider distribution should be associated. This more evident rearrangement between the two structures is also supported by the ideal reorganization of the Mo–S bond; as can be seen in general, C1(n) remains less distorted than C2(n) following the bond distribution in red. The elongated Mo-S bond in gray remains in all the range of Mo atoms per edge for C1(n). Still, a complete trend is observed on C2(2). The presence of these elongated bonds emerges exclusively when $n>7$. The Se-Se dimers formation is exclusively from C2(n) and appears in green. In cyan, the number of shorter Se–Mo bonds remains unaffected for C1(n) as their formation is only through the Mo of the tip. For C2(n), the same behavior is displayed, suggesting that the contracted bonds over the edge are presented in all the range of sizes. Finally, the bond distribution of the Mo-Se interaction in blue is broader again for C2(n) if compared with C1(n) as an effect of the more severe rearrangement. The longest Mo–Se bond in magenta is again symmetrical and unaffected by the increase in size.

The analysis set brings insights into a crucial reaction mechanism that this kind of structure should promote; intriguingly, the bond distributions showed interesting structural properties—that emerge in the atomic region and are significantly modified as the size of the system is altered. This observation hints that the catalytic activity is conditioned on the edge termination and by the size. The study of the role of dimensionality and size-dependent in $MoS_2$ quantum-dots has demonstrated that properties as sulfur bonding strength or hydrogen dissociation are both: size and edge dependent. Besides, through the analysis of STM images, it has been shown that small structures with $n=4$ reveal the formation of spontaneous sulfur vacancies at the edges; an opposite trend has been observed for larger triangles where the vacancies appeared over the entire edge^24^.

### TH-STM analysis

**Figure 5:**
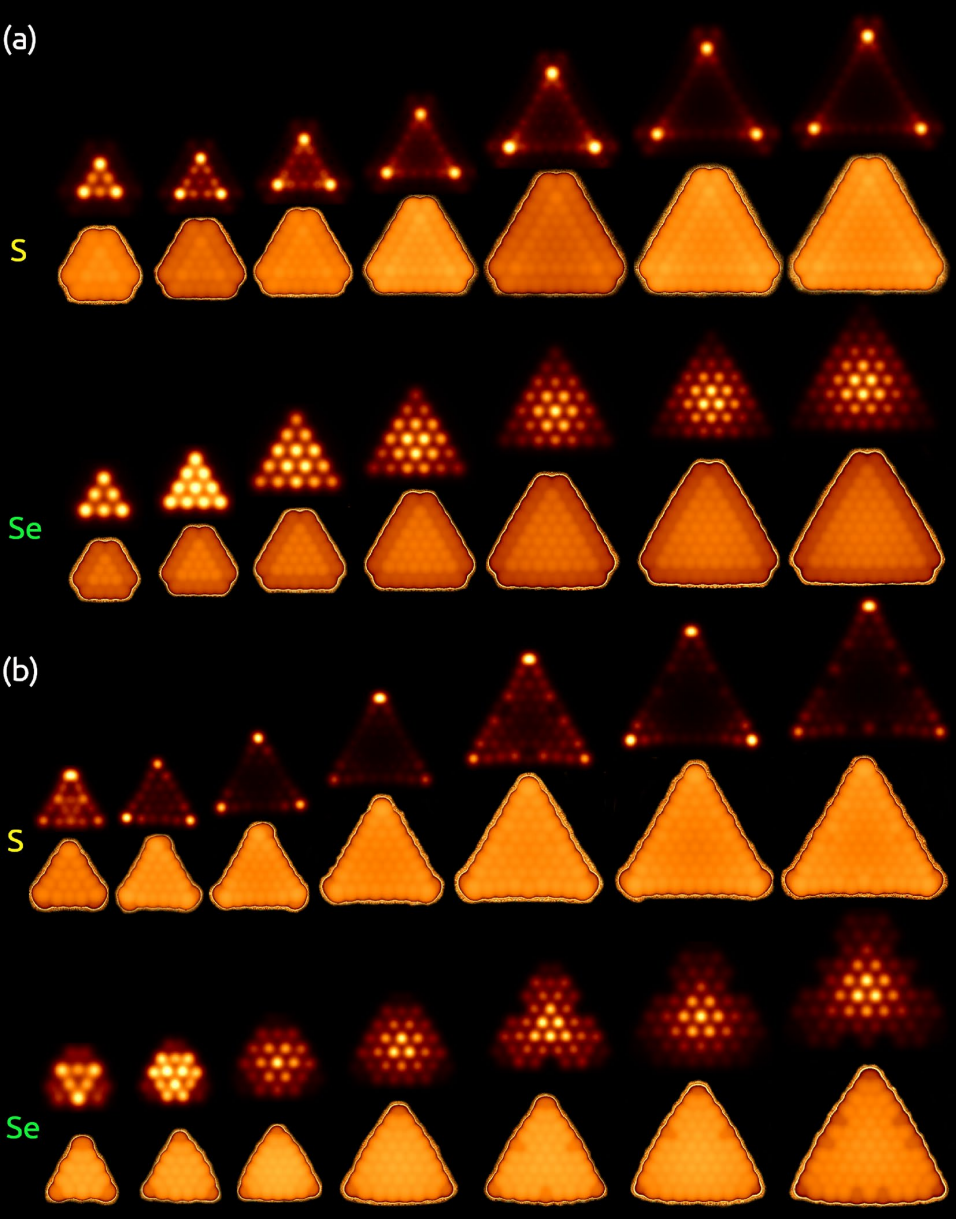
Tersoff-Hamman STM images unravel the atomic-scale details of the C1(n) and C2(n) with n = 4–10. The images presented were obtained by constant height and constant current modes over both faces of the Janus MoSSe quantum dots. (**a**) C1(n), and (**b**) C2(n).

This section focuses on analyzing the morphology of the C1(n) and C2(n) employing the Tersoff-Hamann approximation where the detected current is proportional to the local density of states at the Fermi level where the tip is positioned^33^. The simulated atomically resolved STM images bring insight into the different features shown on both sides of the structure. Figure 5a shows simulated images for C1(n) obtained via constant current utilizing a tunneling current of 5 × 10$^{-5}$ a.u. and in constant height mode with a separation distance from the surface of 3.5 Å.

When using images in constant height mode (upper section in S/Se faces see Fig. 5a,b), the curvature of the structures can be observed due to a greater brightness depending on the side that is analyzed. This contribution is greater at the vertex for the S side, and its appearance is evident from the smaller triangle. For the Se side, the brightness in the central region of the triangle is startled. When $n=4$, the brightness observed over the center of the triangle seems to emulate the metallic edge state observed in the adjacent row of the outermost edge on the $MoS_2$ system^24^. However, with $n>4$, the contribution becomes marked in the center with the highest contribution coming from the Se atom and remains unchanged as the size increases.

For the constant current mode, the images were obtained to decrease curvature’s effect in identifying potential reaction sites (see the lower section in S/Se faces on Fig. 5a,b). Our analysis reveals that the significant contribution arises from the basal plane of both faces in both configurations. Nevertheless, note that for C1, the S side presents a significant contribution coming from the basal plane with a shiny region around the edges; S atoms shifted 0.1 Åin height generates this signal. On the other hand, for the Se side, it can be seen that excluding the Se atoms that decor the edge forming S–Se dimers, a triangular structure emerges that appears to be framed by the border with greater height. The brim contribution of this inner triangle is higher than the edge for all the triangles distribution. These sections are consistent with our previous analysis of geometrical distortion.

The evidence presented is a clear indication that the curvature of the structure is generated towards the side of the sulfur face in both systems. Moreover, the brim regions bring insight into the differential catalytic activity correlated with the regions of higher electron density on both faces. However, an analysis of the charge distribution is vital to assess this critical behavior.

### Electrostatic potential isosurface

**Figure 6:**
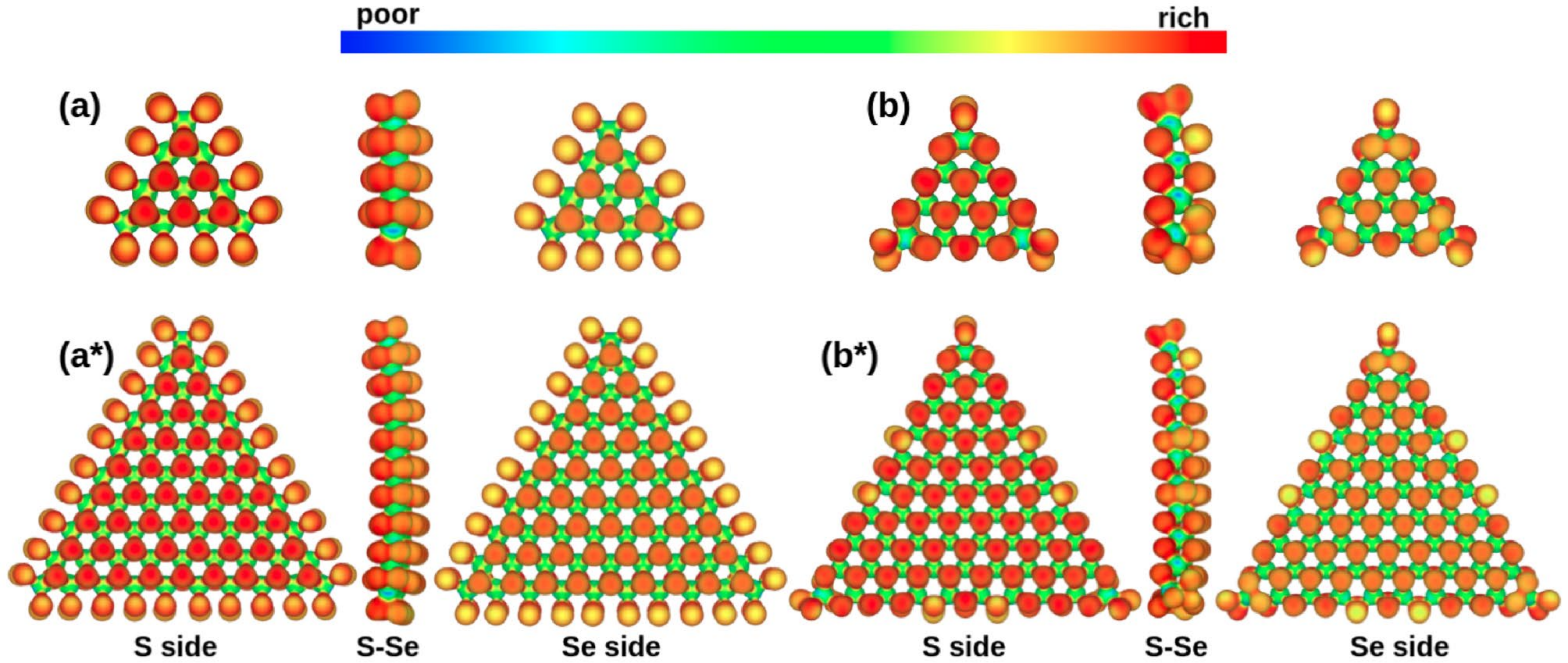
Electrostatic potential isosurface used to visualize electron rich areas on the more stablest structures. (**a**) C1(4), (a*) C1(10), (**b**) C2(4), (b*) C2(10).

Electrostatic potential isosurfaces (EPI) were employed to visualize in real space regions with charge concentration as potential reactive points for the interaction of the quantum dots with target molecules in catalytic reactions. For the representation of EPI, we have used an 0.05 a.u. isovalue and an RGB color code to account for charge accumulation areas in red and charge depletion in blue. The EPI obtained shows a difference in the accumulation of charge on both faces of the Janus structures (see Fig. 6).

In general, the side that contains a more electronegative element—as in this case S, accumulates more charge than the Se face and is denoted by an increment in the red color of the face. Note that both areas account for electron-rich regions compared to the Mo atom at the center of the three layers, whose role is denoted as an electron donor. It is important to mention that for C1(n), the same electronic behavior is observed for both system sizes. Note that the smaller triangles—on both faces enclosed by the outermost Mo atom are more electron-rich than the edges. The differentiated accumulations of electrons observed at the edge can be correlated because S–Se dimers over the edge are bound to one Mo against S/Se atoms towards the center bounded with three Mo each; therefore, these regions are more electron-rich.

For C2(n) (see Fig. 6), the smaller structure presents a uniform electron distribution over the faces; the exceptions are the S–Se dimers at the vertex with a lower concentration, and for the largest model, the new S–Se dimers formed associated with the fourth Mo atom over the edge. This behavior is counterintuitive if compared with the traditional dichalcogenides, whose catalytic activity has been associated with the edges since the basal plane is inactive^34^. A comparison scheme of the projected density of states for isolated S/Se atoms located on edge (without forming dimer), the basal plane, and S–Se dimer is presented in Figs. S2–S4. For an energy window from − 1 to 1 eV, the contributions linked to reactivity are enclosed.

The contributions from the basal plane present localized states below the Fermi level for both species; the broader signal is located behind the Fermi level and moves to the left with the dimer formation. Finally, with the breaking of the dimer and the formation of dangling bonds, the signal reaches its highest value at the Fermi level. This description holds for systems of various sizes. Nevertheless, the role of the curvature shows that as the system size increases, the contribution coming from the atoms on the basal plane diminishes. As the size of the system decreases, the atoms in the basal plane tend to move from their ideal positions since they are more exposed towards the edges; this slight distortion promotes the change in the orbital distribution manifested at the Fermi level. Additionally, the curvature effect observed on both structures emerges from the dimer formation and localization. On C1, all the edges are covered with dimers, while C2 presents their formation primarily on the tips. The electron localization analysis revealed that the metallicity of the system is maintained at the edges and the faces when analyzing the electronic distribution in the different regions containing S/Se atoms so they can be considered reactive sites.

Moreover, our results are consistent with the observations of the Lou et al. group^22^, where the Se/S faces of the MoSSe along with the $MoSe_2$, and $MoS_2$ structures were used as catalytic intermediates for the HER. The efficiency trend obtained through experiment was *SeMoS* (Se face) > *SMoSe* (S face) > $MoSe_2$ > $MoS_2$. Accordingly, their DFT calculations -employing the ML phases of *MoSSe*, $MoS_2$, and $MoSe_2$- for the hydrogen adsorption free energy confirmed that the overpotentials of the basal planes followed the same trend where the lower value was obtained for the Se face of MoSSe^22^.

These results are important to determine the preferential activity of the faces. However, a future study of the energetics of the reactions considering the role of the faces but also de edges is necessary to determine the preferential reaction routes under energy restrictions. These results are beyond the scope of this work and constitute an ongoing project.

## Summary

We have used spin-polarized total energy calculations to study the atomic arrangement of MoSSe quantum dots with different edge terminations and exposure of the border atoms. Our findings show that structures C1, obtained from the Mo$(10{\bar{1}}0)$ termination with S/Se dimers decoring each Mo from the edge and C2 from the S$({\bar{1}}010)$ termination with a zig-zag edge formed by protruding S–Se dimers bonded with two adjacent Mo atoms remain the most stable models; their slight difference in energy implies the coexistence of both structures. Both configurations tend to adopt a simple-concave morphology. However, C1 is curved to a lesser degree than C2. The geometrical distortion analysis reveals that the bond distribution in C1 is uniform as the S–Se dimers of the edges tend to stabilize it; the opposite trend is observed in C2. The bond distributions showed that structural properties are modified as the size of the system is altered. This observation hints that the catalytic activity is conditioned on the edge termination and by the size.

TH-STM constant current images showed the absence of metallic states over the edge for both faces; in contrast, both configurations showed a greater contribution coming from a triangular section formed by S/Se atoms enclosed by the S–Se dimers over the edge. The constant height images also confirmed these contributions.

Finally, hypothetical reactive points were obtained by the EPI analysis. The results show that the S side exhibits a much higher electron density concentration than the Se side. Moreover, C1 displays an electronic distribution that points toward the preferential activity of the basal plane; the same trend emerges for C2, where the full-face displays a uniform distribution except for the S–Se dimers that emerge by size effects. These results are relevant to define the preferential activity of the faces. However, a future investigation to study energetics on model reactions is mandatory to define the preferential reaction routes.

## Methods

The stability of MoSSe quantum dots as a function of their size and termination has been analyzed by spin-polarized total-energy calculations through density functional theory as implemented in the Vienna ab initio simulation package^35–39^. Exchange-correlation energies were treated within the generalized gradient framework (GGA) with a Perdew-Burke-Ernzerhof (PBE) functional^40^. Valence states were represented by the frozen core approach by the projected augmented waves method^41^. The electronic states of the pseudo-wave function were modeled with an expansion of plane waves with an optimized cutoff energy of 500 eV. The energy corrections for the long-range interactions were taken into account by the Grimme-D3 method^42^. The supercell method was applied to isolate and impose periodic conditions between neighboring images of the MoSSe quantum dots with a separation distance of 13 Å. The force and energy criteria employed for the structural optimization were achieved when differences were lower than 0.01 eV/Å and 1 × 10$^{-4}$ eV. To sample the Brillouin zone, we employed the Monckhorst-Pack scheme^43^. A gamma-centered k-point and 12 × 12 × 1 k-points grids were used for the quantum dots and the MoSSe ML, respectively. For the SFE analysis, the Mo bulk was calculated in a BCC configuration with a lattice parameter of 3.12 Å and a 12 × 12 × 12 k-point grid. The alpha-S and Se bulks were modeled as eighth-ring members in a Monoclinic configuration with lattice parameters (for alpha-S/Se) a = 10.46/12.93 Å, b = 12.86/8.08 Å, and c = 24.48/9.23 Å. Accordingly, their k-point grids were 3 × 3 × 2 and 3 × 4 × 4.

### Structural models

As “Structural configurations” contains the structural features of the relaxations, here we present the details of the four proposed triangular models (see Fig. 1). As mentioned earlier, the quantum dots were built taking the number of Mo per edge (n) as a reference to set the size of the system.

For a given n value: C1, C2, and C3 contain the same total number of Mo atoms, whereas C4 has 2n additional Mo Atoms. The number of S and Se atoms is the same in each configuration as imposed by symmetry. However, this value changes between configurations for a fixed n. Table S1 contains the total number of species for each model in all the ranges of sizes.

### Formation energy formalism

To study the stability of different sizes and terminations of MoSSe quantum dots, we have employed the surface formation energy formalism (SFE). Our analysis takes as reference the $MoS_2$ bulk to compare the stability of the layered structures $1H-MoS_2$ against 1H-MoSSe and different size MoSSe quantum dots. The SFE per atom considers the chemical potentials of each atomic species with the general formula $Mo_xS_ySe_z$.

1 $$\begin{aligned} SFE(\mu _{Mo},\mu _{Se},\mu _{S})=\frac{E_{Mo_xS_ySe_z}-x\mu _{Mo}-y\mu _{S}-z\mu _{Se}}{x+y+z} \end{aligned}$$

where $E_{Mo_xS_ySe_z}$ is the total energy of the quantum dot, $\mu _{X}$ corresponds to the chemical potential of the X species, and x, y, and z are the number of Mo, S, and Se atoms.

Thermal equilibrium between the bulk, vacuum, and molecular structure has been considered to establish the following correlation among chemical potentials

2 $$\begin{aligned} \mu _{Mo}^{Bulk}+2\mu _{S}^{Bulk}+\Delta H_f=\mu _{MoS_2}=\mu _{Mo}+2\mu _{S} \end{aligned}$$

where $\Delta H_f$ corresponds with the formation enthalpy of the reference—this is the ${MoS_2}^{Bulk}$ and the chemical potentials of the X species are inter-correlated with their corresponding bulk phases via:

3 $$\begin{aligned} \mu _{X}\le \mu _{X}^{Bulk} \end{aligned}$$

since

4 $$\begin{aligned} \mu _{MoS_{2}}^{Bulk}=\mu _{Mo}+2\mu _{S} \end{aligned}$$

The SFE can be rewritten considering Eqs. (1) and (4) as follows.

5 $$\begin{aligned} SFE(\mu _{Se},\mu _{S})=\frac{E_{Mo_xS_ySe_z}-x\mu _{MoS_2}^{Bulk}+\mu _{S}(2x-z)-y\mu _{Se}}{x+y+z} \end{aligned}$$

## Supplementary Information

Supplementary Information.
