# Supplementary material for: The effect of shape and size in the stability of triangular Janus MoSSe quantum dots
Source: Sci Rep. 2021 Oct 26;11:21061. doi: 10.1038/s41598-021-00287-6 (PMC8548305; doi:10.1038/s41598-021-00287-6)
Supplement: Supplementary file 1 — Supplementary Information. [file 41598_2021_287_MOESM1_ESM.pdf]

The effect of shape and size in the stability of triangular Janus MoSSe quantum dots (Supplementary Section)

J.I. Paez-Ornelas[1,2,*], R. Ponce-Perez[2], H. N. Fernández-Escamilla[2], D.M. Hoat[3,4], E.A. Murillo-Bracamontes[2], Ma Guadalupe Moreno Armenta[2], Donald H. Galván[2], and J. Guerrero-Sánchez[2,*]

[1] Centro de Investigación Científica de Educación Superior de Ensenada, Ensenada Baja California, 22800, México
[2] Centro de Nanociencias y Nanotecnología, Universidad Nacional Autónoma de México, Apartado Postal 14,Ensenada Baja California, Código Postal 22800, México
[3] Institute of Theoretical and Applied Research, Duy Tan University, Hanoi 100000, Viet Nam
[4] Faculty of Natural Sciences, Duy Tan University, Da Nang 550000, Viet Nam
*josepaez@cicese.edu.mx,*guerrero@ens.cnyn.unam.mx

Stability of the models

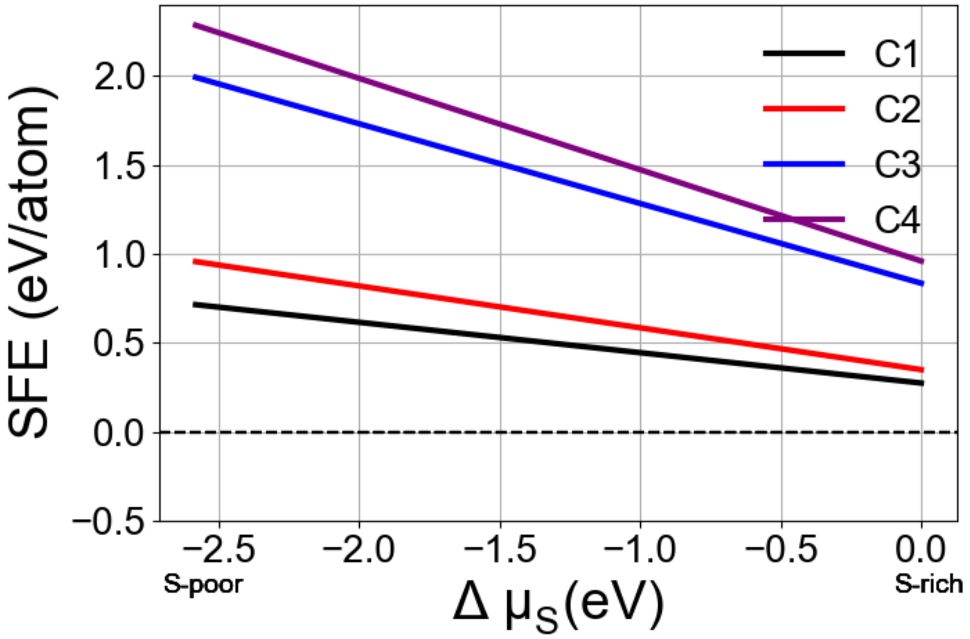

Figure S1. SFE energies of triangular configurations as a function of Sulfur chemical potential. Rich/poor growth conditions stand for bulk-sulfur/MoS_2 formation enthalpy.

Structural models

Details of the structural models. The size of the systems is given by the number of Mo atoms on edge (n). Table S1 details the number of atoms by species for each configuration based on the four structures presented.

C1	C2	C3	C4	n
10/18/18	10/15/15	10/6/6	18/10/10	4
15/25/25	15/21/21	15/10/10	25/15/15	5
21/33/33	21/28/28	21/15/15	33/21/21	6
28/42/42	28/36/36	28/21/21	42/28/28	7
36/52/52	36/45/45	26/28/28	52/36/36	8
45/63/63	45/55/55	45/36/36	63/45/45	9
55/75/75	55/66/66	55/45/45	75/55/55	10
**Mo/Se/S atoms**				

Table S1. The number of atoms of each species (Mo/S/Se) on each configuration for all the distribution of sizes (n=4-10).

Projected density of states (pDOS)

The projected pDOS for the S/Se atoms on the basal plane, the edge S/Se atoms without the dimer formation, and the edge S-Se dimer are presented. Three configurations with different sizes are plotted to analyze the size effect on the QD electronic behavior.

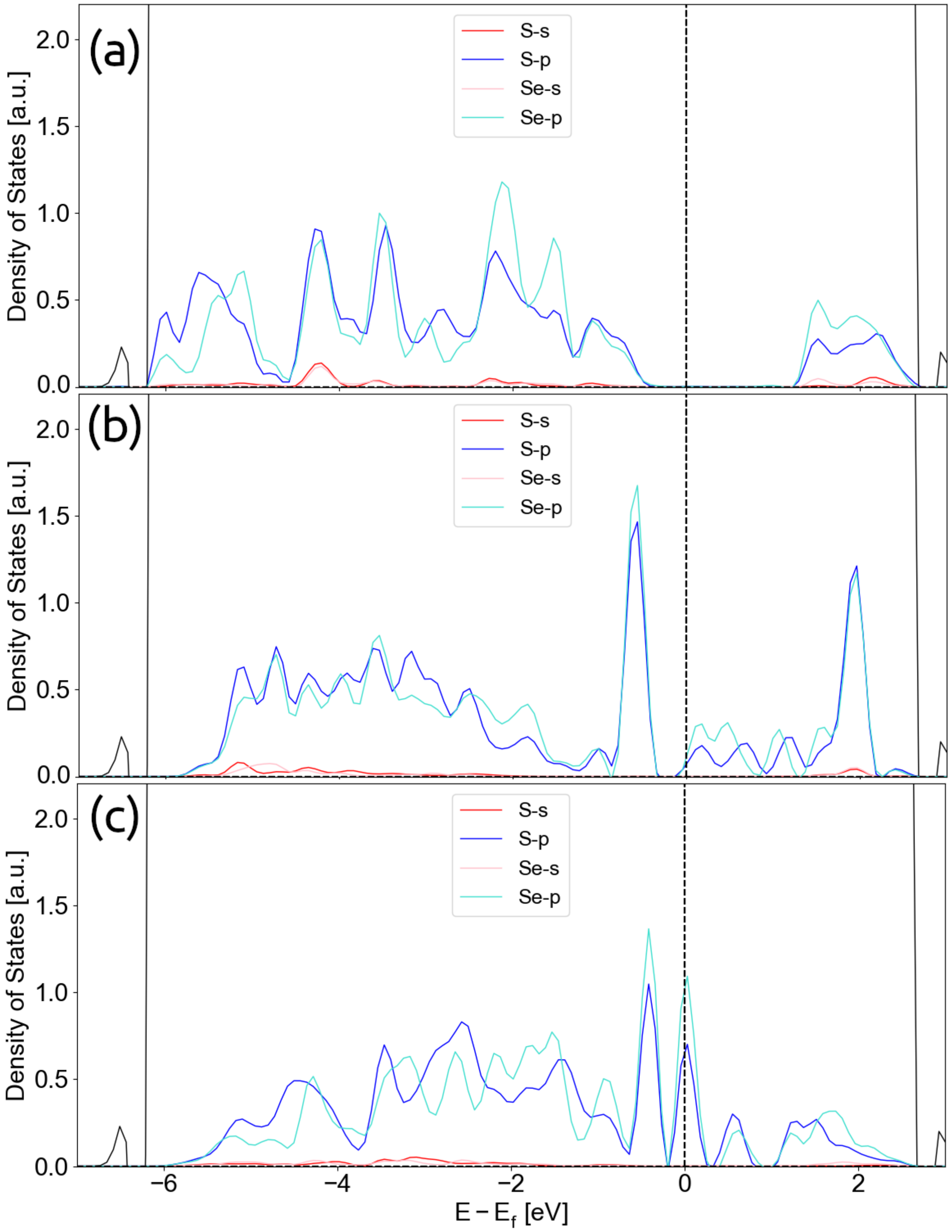

Figure S2. Projected DOS for C2(10). (a) S/Se from the basal plane, (b) S-Se dimer, and (c) non-bonded edge S-Se. Fermi level set at zero energy.

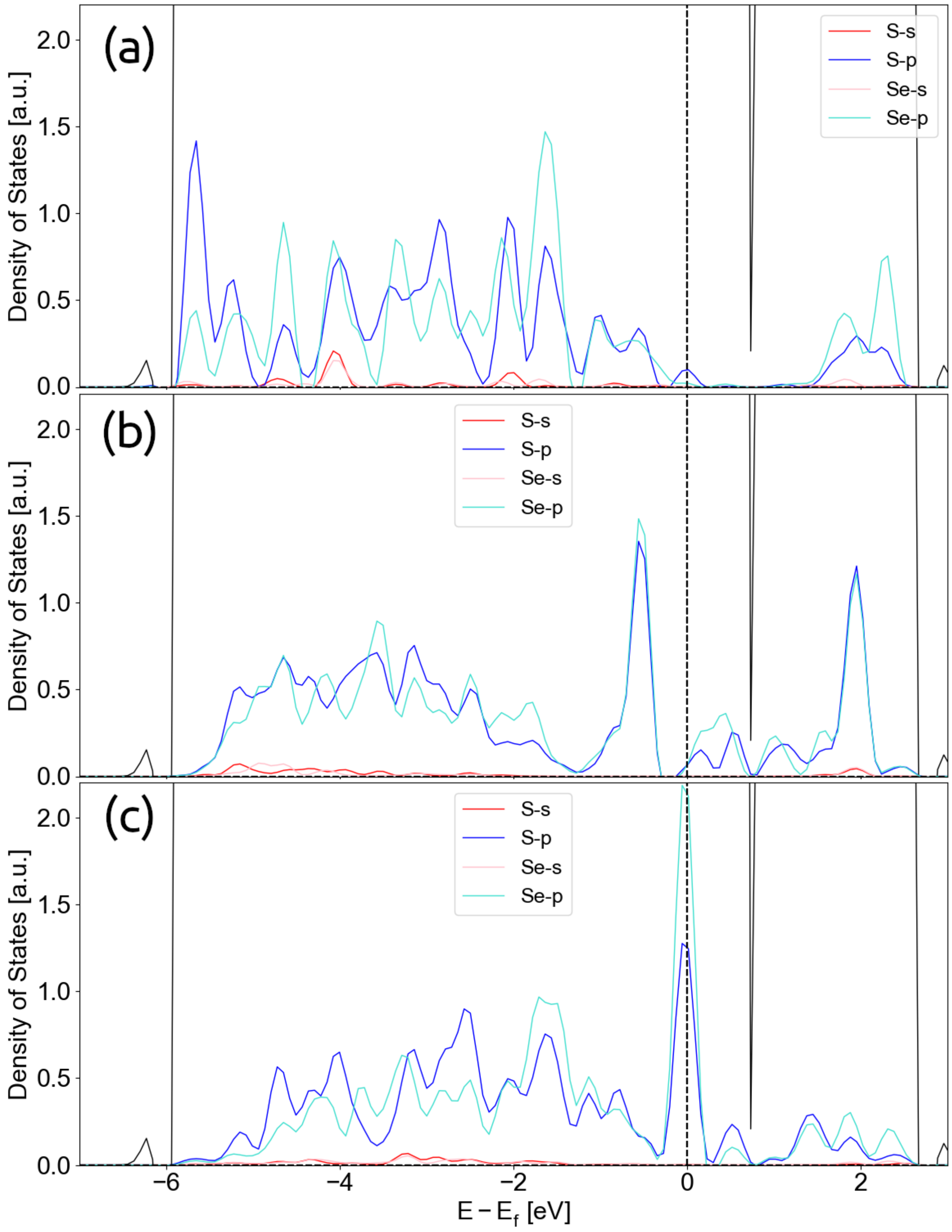

Figure S3. Projected DOS for C2(6). (a) S/Se from the basal plane, (b) S-Se dimer, and (c) non-bonded edge S-Se. Fermi level set at zero energy.

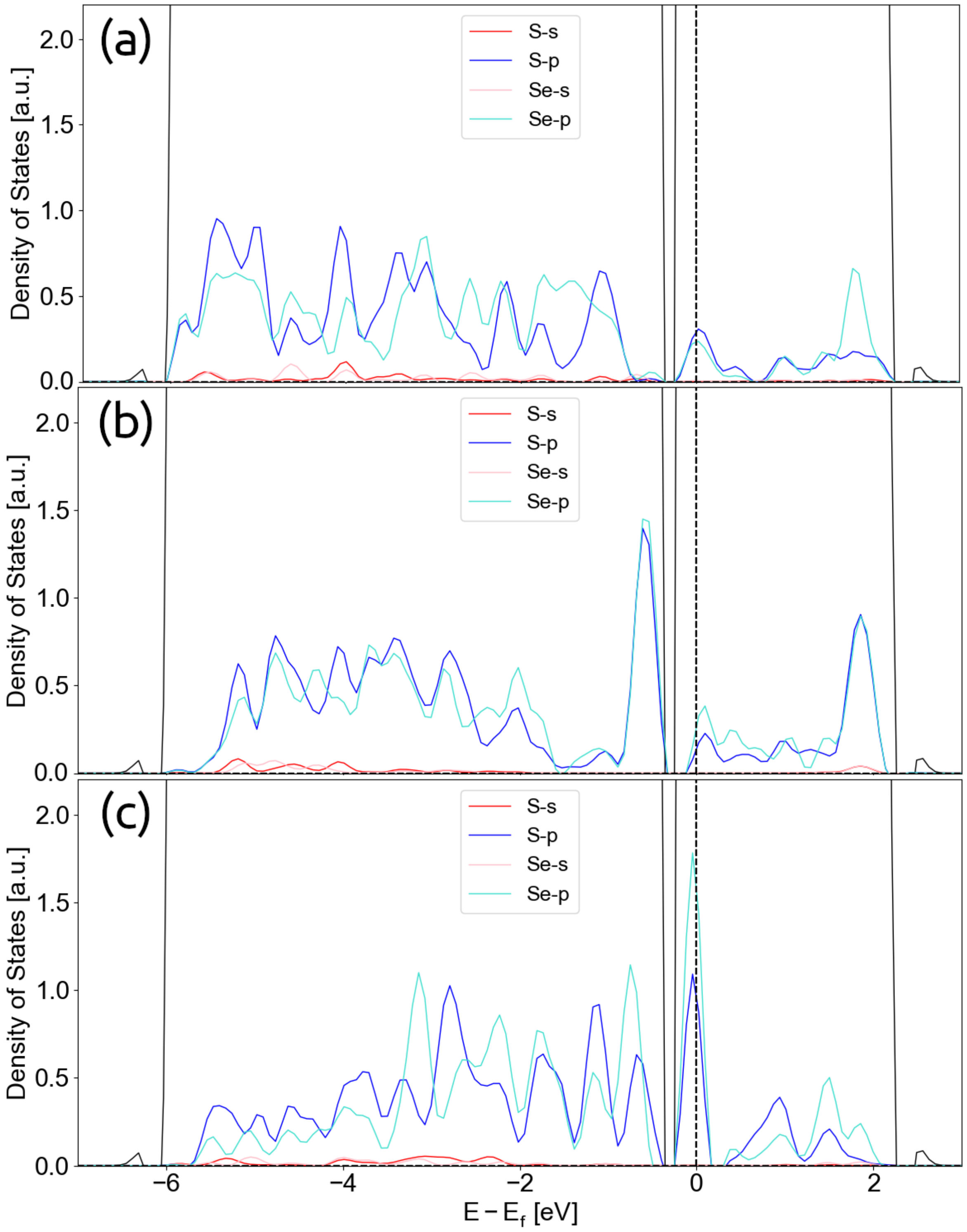

Figure S4. Projected DOS for C2(4). (a) S/Se from the basal plane, (b) S-Se dimer, and (c) non-bonded edge S-Se. Fermi level set at zero energy.
